# Parodontgel® on Wound Healing and Patient-Reported Outcome Measures (PROMs) after Tunneled Coronally Advanced Flap (TCAF)

**DOI:** 10.1155/2024/5571545

**Published:** 2024-01-25

**Authors:** Leonardo Mancini, Vincenzo Mancini

**Affiliations:** ^1^Center for Clinical Research and Evidence Synthesis in Oral Tissue Regeneration (CRITERION), Boston, Massachusetts, USA; ^2^Clinic of Reconstructive Dentistry, Centre of Dental Medicine, University of Zurich, Zurich, Switzerland; ^3^Private Practice, Avezzano, Italy

## Abstract

The adjunctive use of healing gels following periodontal plastic surgery is not common in clinical practice, and no definitive benefits have yet been demonstrated. *Case Presentation*. A 33-year-old male patient with a central lower incisor class RT1 recession sought treatment due to sensitivity and dissatisfaction with the appearance of his smile. The patient had no history of periodontal disease; however, he was under orthodontic treatment contributing to the gingival recession and irregular gum contours. *Treatment*. The patient underwent two sequential surgical procedures. Initially, an apically repositioned flap (APF) was performed to correct the frenulum reducing flap tension and improving the gum line aesthetics. Subsequently, after 8 weeks, a tunneled coronally advanced flap (TCAF) was executed to further refine the gum contours and achieve root coverage. *Postoperative Healing Protocol*. To enhance the healing process and alleviate postoperative discomfort, a healing gel containing hyaluronic acid as the active molecule was applied to the surgical sites. The gel was expected to reduce pain perception and minimize the need for painkiller intake during the critical first week of recovery. The patient was asked to fill a pain chart for the initial 7 days, recording pain levels on a visual analogue scale (VAS 0-10) and the number of paracetamol tablets taken as painkillers. *Results*. After both the APF and TCAF surgeries, the patient reported pain levels with a mean VAS score of 4.33 and 4.25, respectively. The painkiller intake during the first week was noted to be 3 tablets for the APF and 2 tablets for the TCAF. Notably, the application of the healing gel with hyaluronic acid did not cause any adverse reactions, indicating its potential safety and efficacy in this context. *Conclusion*. The application of a healing gel containing hyaluronic acid after periodontal plastic surgery showed promising results in reducing postoperative pain and the need for painkillers during the initial week of recovery. However, further investigations through randomized clinical trials are required to establish the potential benefits and broader applicability of such healing gel applications in the context of periodontal plastic surgery.

## 1. Introduction

The term gingival recession describes a common condition affecting a significant segment of the population [1, 2]. The use of autogenous connective tissue grafts (CTGs) has been shown to be the most effective and reliable method of promoting root coverage [3–5]. However, it is always associated with a second surgical site which can increase pain and discomfort during the first 7 days of healing [6, 7]. Therefore, several soft tissue substitutes were introduced on the market to overcome this situation and reduce the so-called patient-reported outcome measures (PROMs).

Collagenated matrixes and acellular dermal matrixes are the ones with more literature and evidence; however, these soft substitutes do not perform as the gold standard [8]. This means showing always or in 95% of the cases a complete root coverage. Consequently, CTG is the standard and mostly in several complicated cases as RT2 and RT3 according to the latest classification [7, 9].

The harvesting of CTG from the palate is one of the common techniques used to obtain autogenous soft tissue ready to be grafted on the required site. In several situations, however, there are some anatomical issues related to the palatine artery which can be led during the harvesting procedure causing severe bleeding and increasing the surgical time which is another crucial factor in order to minimize patient discomfort [10].

The healing period and the first days of healing are crucial for the stability of the graft and the readaptation of the flap or tissues around the treated site [11]. During this period, patients showed swelling, edema, pain, and some minor bleeding in the first 24 hours. To overcome these issues, reducing PROMS and enhancing healing, several companies have released healing gels capable to provide at the same time protection and boost for the healing [12–14]. Most of them are made of several active molecules that act on fibroblast maturation and promote reepithelialization, retaining water molecules and creating an environment conducive to cell survival. The aim of this case report is to show the possible application and impact that those types of gels have on PROMs and on tissue healing during the first week after surgery.

## 2. Case Report

The present case report followed CARE checklist and CARE guidelines, in accordance with the Declaration of Helsinki updated in 2013 [15]. A written informed consent was obtained before treatment and for the publication of the present case report.

**Figure 1:**
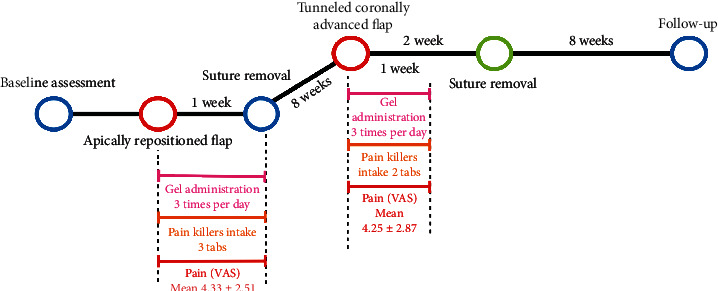
The timeline depicts the baseline assessment, the apically repositioned flap (in red), suture removal, soft tissue healing, and TCAF (in red), reporting pain killer's intake, gel administration, and pain levels for each postoperative week.

**Figure 2:**
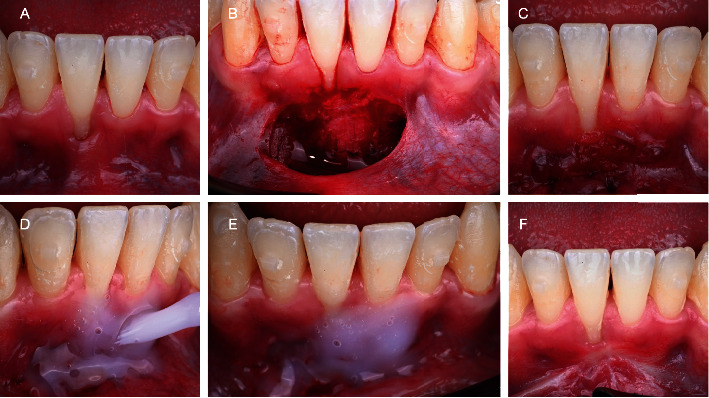
Apically repositioned flap and gel application directly after surgical intervention.

**Figure 3:**
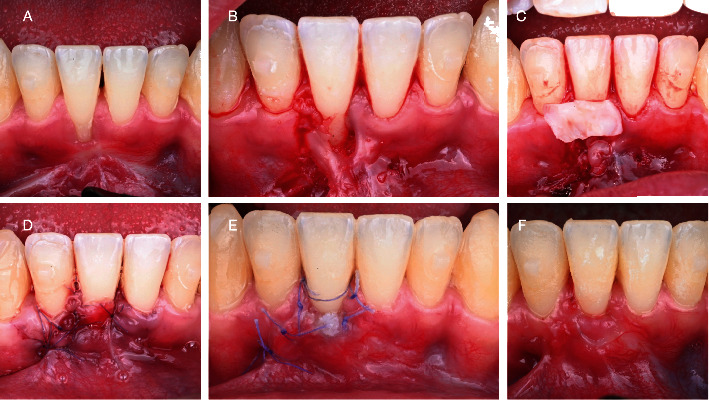
Tunneled coronally advanced flap step by step: (A) baseline after 8 weeks from the apically repositioned flap; (B) incision; (C) soft tissue grafting; (D) suturing phase; (E) after 7 days; (F) at 4 weeks.

A young 33-year-old male presenting healthy systemic conditions and good oral health, after orthodontic treatment with aligners (Invisalign, Align Technology, Tempe, Arizona, USA) for aesthetic purposes, was complaining about the presence of a gingival recession in zone 4.1. The tooth did not present any clinical attachment loss at the mesial and distal aspect; moreover, soft tissue phenotype was thick. According to the latest classification, the defect was classified as a RT1 [16]. However, the presence of a thick frenulum at the apical portion of the site did not allow to perform directly a coronally advanced flap. Therefore, the patient was subjected to 2 interventions in an interval of 2 months. As mentioned on the timeline (Figure 1) there was a first apically repositioned flap that allowed to remove tension on the site (Figures 2(A)–2(F)). After 8 weeks, a tunneled coronally advanced flap (TCAF) as described by Barootchi and Tavelli was applied combined with a CTG harvested from the palate (Figures 3(A)–3(F)) [17].

The TCAF technique for treating isolated RT1-RT2 gingival recessions involved elevating one trapezoidal surgical papilla and executing a single vertical incision. A slightly divergent vertical incision was performed using a minicrescent knife (Surgistar Micro 25°, Surgistar, Vista, California, USA). A minicrescent knife was also utilized to execute the intracellular incision on the treated site and on the tooth adjacent to the papilla that was being preserved. To achieve tension-free flap advancement, one more tooth after the recession defect (not on the site adjacent to the vertical incision) was tunneled. The midfacial aspect of the tooth was elevated with tunneling knives (Sharptome, Surgical Specialties American Dental Systems, Vaterstetten, Germany) and TNK1 and TNK2 (Hu-Friedy, Chicago, USA), while the surgical papilla was incised and elevated in a split-thickness manner with a miniblade (miniblade no. 67, Salvin Dental Specialties, Charlotte, North Carolina, USA).

The flap was then released with a 15C blade (Swann-Morton, Sheffield, UK) from the area in which the surgical papilla and the vertical incision were performed and was further completed by introducing curved tunneling knives (Hu-Friedy, Chicago, USA) from the sulcus of the tooth with the intact papilla. The flap was considered tension-free only when it was able to passively reach a level approximately 2 mm coronal to the cementoenamel junction. The anatomical papilla that was incised was then deepithelialized with a miniblade (miniblade no. 67, Salvin Dental Specialties, Charlotte, North Carolina, USA), while the other papilla was gently detached from the interproximal bone and mobilized with a papilla elevator instrument PH26M (Hu-Friedy, Chicago, USA).

After mechanical and chemical root conditioning with 24% ethylenediaminetetraacetic acid (EDTA) for 2 min and rinsing with sterile saline, a connective tissue graft (CTG) obtained from the palate as a free gingival graft and extraorally deepithelialized was inserted underneath the flap and tunneled below the nonincised papilla. The graft was then sutured to the deepithelialized anatomical papilla with a simple interrupted suture (6/0 resorb, Sweden & Martina, Padova, Italy). Further stabilization of the graft was obtained with simple interrupted sutures engaging the periosteum and sling sutures around the tooth (6/0 resorb, Sweden & Martina, Padova, Italy). The flap was then coronally advanced and sutured with sling sutures from the incised papilla to the tunneled papilla and from the incised papilla to the papilla of the adjacent tooth not involved in the flap. The vertical incision was then approximated to the adjacent soft tissue with simple interrupted sutures. Figures 3(A)–3(F)) illustrate the execution of the TCAF.

This technique seems to be minimally invasive; indeed, only one papilla is deepithelialized whereas the other is tunneled to maintain stable tissues and support for the graft underneath. The use of a single releasing incision mesial or distal to the tooth allowed to release and easily advance the flap in order to cover the CTG underneath ensuring a blood supply to the graft. The main important aspect of this procedure is the split thickness of the flap, the stabilization of the graft, and finally the stabilization of the flap with slicing sutures on the papillae [11].

Intraoral scans were taken before and after root coverage procedure to observe the gain in volume, thickness, and root coverage achieved. A novel 3D software (GOM Inspect, Carl Zeiss S.p.A., Salerno, Italy) was used to superimpose the scans and evaluate all the parameters described above. The 3D analysis consists of a first phase of superimposition and then the delimitation of a region of interest on top of the treated site. This method is extensively used in the dental field and mainly for soft tissue augmentation procedures as reported in several studies [6, 18, 19].

**Figure 4:**
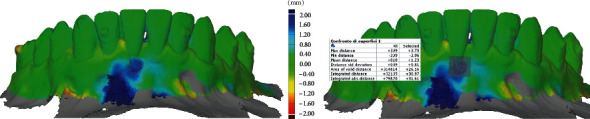
Volumetric analysis showing the treated site where an increase in thickness was observed (blue area).

**Figure 5:**
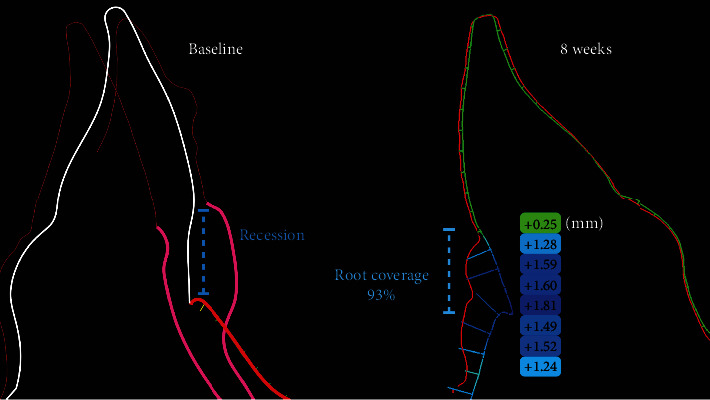
Linear changes from baseline to 8 weeks at the buccal aspect show a root coverage of 93% and a soft tissue thickness ranging from 0.25 to 1.81 mm.

Figure 4 shows the superimposed model and the analysis performed with a squared region of interest starting for the cement-enamel junction (CEJ) considering the mesial and distal papilla through the apical portion of the exposed root. At 8 weeks after the TCAF procedure the mean gain obtained was of 1.23 mm with a maximum of 3.75 mm and a minimum of 0.25 mm, moreover, using a cross-section of the scans and highlighting 8 points at 1 mm from each other it was possible to observe how the CTG underneath was crucial for the increase root coverage (Figure 5).

### 2.1. Gel Administration and PROMs

**Figure 6:**
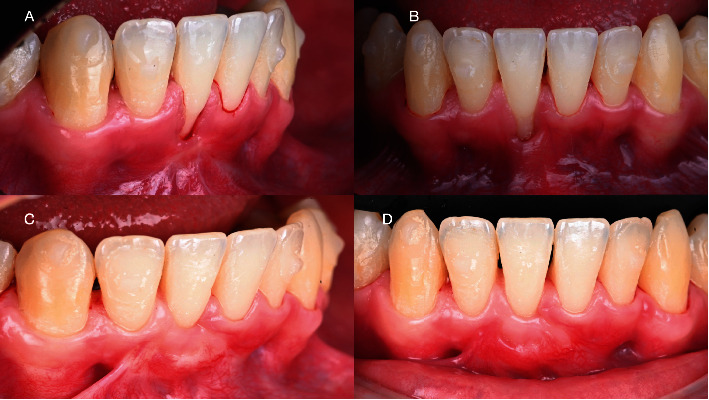
(A) Baseline lateral perspective; (B) baseline frontal perspective; (C) lateral view at the 8-week follow-up; (D) frontal view at the 8-week follow-up.

After performing both surgeries, a gel (Biorepair Parodontgel, Coswell Spa, Bologna, Italy) with hyaluronic acid and other active molecules (Biorepair Plus Parodontgel® Protezione Gengive: aqua, zinc hydroxyapatite (microRepair®) 20%, glycerin, sorbitol, hydrated silica, silica, cocamidopropyl betaine, cellulose gum, aroma, lactoferrin, sodium myristoyl sarcosinate, sodium methyl cocoyl taurate, Hamamelis virginiana leaf extract, Spirulina platensis extract, Calendula officinalis flower extract, zinc PCA, sodium hyaluronate, tocopheryl acetate, retinyl palmitate, sodium saccharin, phenoxyethanol, benzyl alcohol, sodium benzoate, potassium sorbate, limonene, and CI 77891) was applied directly on the wound 3 times per day for 7 days (Figures 2(D) and 2(E)). The patient was carefully instructed to not pull the lips or scrape the gingiva while administrating the gel for the entire healing period. Painkiller intake and pain were recorded for the entire week. To report pain, a visual analog scale (VAS) from 0 to 10 was used, and every day, the patient had to fill a precompilated chart with the VAS and the number of tablets of painkillers used. As shown in Figure 1, the pain killer intake during the healing period was 3 tablets for the first surgery and 2 tablets after TCAF, while the pain perception showed a mean of 4.33 and 4.25, respectively. During the entire healing period, the application of the healing gel did not show any adverse effect conversely showing reduced edema swelling and patient discomfort.  Figure 6 shows the baseline and 8-week follow-up results.

## 3. Discussion

The tunneled coronally advanced flap (TCAF) is a surgical technique used in periodontal plastic surgery to treat gingival recessions. It is an advanced method that involves creating a tunnel-like incision in the gum tissue, which is then used to cover exposed tooth roots and correct the recession. TCAF is preferred for its aesthetic results and minimal tissue trauma trying to reduce invasiveness [11, 17]. Pain and discomfort are common concerns after any surgical procedure, including periodontal plastic surgery. Patients often experience mild-to-moderate pain during the initial healing period, which typically lasts for a few days to a week [20]. Painkiller intake is another aspect to consider after periodontal plastic surgery. Patients may be prescribed pain medication, such as nonsteroidal anti-inflammatory drugs (NSAIDs) or analgesics, to manage postoperative pain [21, 22]. The amount and frequency of painkiller intake depend on the individual's pain tolerance and the extent of the surgery [23]. It is essential for patients to follow the prescribed pain management regimen and report any severe or persistent pain to their periodontist. Proper postoperative care, including rest, maintaining oral hygiene, and avoiding certain foods, can also help minimize pain and promote healing. Although in this case report a minimally invasive procedure such as TCAF was used, individual responses to surgery can vary. Some patients may experience minimal discomfort and require little to no pain medication, while others may need more assistance to manage their pain during the healing process [23, 24]. In order to overcome and reduce pain issues related and pain killer's intake, the adjuvant of a healing gel as mentioned in this case report might help in reducing PROMs and therefore facilitating minimized postoperative sequelae. In literature, several gels based on aloe vera or chlorhexidine were used after tooth extraction or after periodontal regeneration procedures with some doubt on the possible toxicity or inefficacy of the molecules on tissue maturation [25, 26].

On the potential role of hyaluronic acid in tissue healing, data are available with a positive result when used on the palate after CTG harvesting showing reduced pain and pain killer intake [12, 14, 27]; however, the topical application directly on the surgical site is still missing, and therefore, this case report might help to move a step forward through the improvement of patient experience after periodontal plastic surgeries with the application of healing gel after surgery and for the first 7 days of healing.

## 4. Conclusion

The application of a healing gel containing hyaluronic acid directly on the surgical site after suturing has demonstrated promising results in reducing pain and painkiller intake, leading to a potentially improved surgical experience for the patient. Moreover, this intervention seems to contribute to reduced patient-reported outcome measures (PROMs), indicating a positive impact on patient satisfaction and overall well-being following periodontal plastic surgery. However, it is crucial to note that these findings are based on a single case report, and further evidence is needed to validate the efficacy and safety of this approach. The absence of any adverse effects in the presented case report is encouraging, suggesting that the application of the gel was well tolerated by the patient. Nonetheless, it is essential to conduct future randomized clinical trials to establish the effectiveness of this gel compared to a control or placebo group. Randomized controlled trials provide a higher level of evidence and can confirm whether the observed benefits are consistent and statistically significant across a broader patient population.

## Data Availability

The data that support the findings of this study are available from the corresponding author (LM) upon reasonable request.
